# Book Notices

**Published:** 1870-01

**Authors:** 


### Book Notices.

---

A Treatise on Intraocular Tumors, from Original Clinical Observations and Anatomical Investigations. (With one Chromo-Lithographic and fifteen Lithographic Plates. By H. KNAPP, M.D., late Professor of Ophthalmology and Surgeon to the Ophthalmic Hospital, in Heidelberg. Translated by S. COLE, M.D., of Chicago. New York: W. Wood & Co., 61 Walker St. 1869.

We have received a copy of this work from the translator. It is an elegantly publiseed volume of over 300 pages. The paper, type, and illustrations are all good. The subjects of which it treats are not of very frequent occurrence, generally obscure in their origin, and, consequently, of difficult diagnosis. The work will be of special interest to the ophthalmologist and of use to the general practitioner.

---

The Pathology of Bright's Disease. By WM. B. LEWIS, M.D., Lecturer on Renal Pathology, in the Medical Department of the University of the City of New York; Microscopist of Charity Hospital, etc. With illustrations. New York: Turner & Migmed. 1869.

This is an interesting pamphlet of 29 pages, containing a brief history of renal pathology, and a clear statement of the present status of medical opinions, in relation to Bright's disease.

---

Minutes of the First Annual Meeting of the Nebraska State Medical Society, held in Nebraska City, June 1st and 2d, 1869.

This is a pamphlet of 24 pages, containing the record of proceedings of the annual meeting, embracing brief reports on Surgery, by Drs. S. D. Mereer and N. B. Larsh, and on Obstetrics, by Dr. James H. Peabody, and the constitution and bye-laws of the Society. The proceedings indicate a good degree of enterprise and talent in the profession of this new State. The officers for the present year are, *President*, James H. Peabody; *First Vice-President*, N. B. Larsh; *Second Vice-President*, F. Renner; *Corresponding Secretary*, J. C. Denise; *Permanent Secretary*, S. D. Mereer; *Treasurer*, D. W. Hershey.

---

Archives de Physiologie, Normale et Pathologique. Published by Brown-Séquard, Charcot, and Vulpian, of Paris, France. This very valuable periodical for November and December is before us, filled with its usual variety of interesting articles.

